# Structural Basis of pH Dependence of Neoculin, a Sweet Taste-Modifying Protein

**DOI:** 10.1371/journal.pone.0126921

**Published:** 2015-05-26

**Authors:** Takayuki Ohkubo, Minoru Tamiya, Keiko Abe, Masaji Ishiguro

**Affiliations:** 1 Department of Applied Life Sciences, Niigata University of Pharmacy and Applied Life Sciences, Higashijima, Akiha-ku, Niigata, Japan; 2 Department of Applied Biological Chemistry, Graduate School of Agricultural and Life Sciences, The University of Tokyo, Yayoi, Bunkyo-ku, Tokyo, Japan; University of Tsukuba, JAPAN

## Abstract

Among proteins utilized as sweeteners, neoculin and miraculin are taste-modifying proteins that exhibit pH-dependent sweetness. Several experiments on neoculin have shown that His11 of neoculin is responsible for pH dependence. We investigated the molecular mechanism of the pH dependence of neoculin by molecular dynamics (MD) calculations. The MD calculations for the dimeric structures of neoculin and His11 mutants showed no significant structural changes for each monomer at neutral and acidic pH levels. The dimeric structure of neoculin dissociated to form isolated monomers under acidic conditions but was maintained at neutral pH. The dimeric structure of the His11Ala mutant, which is sweet at both neutral and acidic pH, showed dissociation at both pH 3 and 7. The His11 residue is located at the interface of the dimer in close proximity to the Asp91 residue of the other monomer. The MD calculations for His11Phe and His11Tyr mutants demonstrated the stability of the dimeric structures at neutral pH and the dissociation of the dimers to isolated monomers. The dissociation of the dimer caused a flexible backbone at the surface that was different from the dimeric interface at the point where the other monomer interacts to form an oligomeric structure. Further MD calculations on the tetrameric structure of neoculin suggested that the flexible backbone contributed to further dissociation of other monomers under acidic conditions. These results suggest that His11 plays a role in the formation of oligomeric structures at pH 7 and that the isolated monomer of neoculin at acidic pH is responsible for sweetness.

## Introduction

Plants produce a variety of sweet tasting compounds that are classified as low molecular weight non-proteins or high molecular weight proteins. Sweet proteins have been discovered from a variety of natural sources and to have a variety of action modes. Thaumatin [1] and monelin [2] were reported in 1972 and two decades later, mabinlin [3] was reported in 1993 and brazzein [4] was reported in 1994.

Taste-modifying proteins miraculin [5] and neoculin [6] were identified in 1968 and 1990, respectively. Miraculin, which belongs to the Kunitz-type trypsin inhibitor protein family, was isolated from the fruit of *Richardella dulcifica* (family: Sapotaceae) in West Africa. It was identified as a homodimer of a protein consisting of 191 amino acids connected with a disulfide bond at Cys138 [7]. Neoculin was isolated from the fruit of *Cuculigo latifolia* (family: Hypoxidaceae) in Malaysia and was shown to form a heterodimer with the acidic subunit (NAS) consisting of 113 amino acids and the basic subunit (NBS) consisting of 114 amino acids joined by two disulfide bonds [8]. The two neoculin subunits are related to the protein family of mannose-binding lectin.

Although neoculin and miraculin show no amino acid sequence similarity, these two proteins have taste-modifying properties. Neoculin is slightly sweet at pH 7 and very sweet at lower pH while miraculin is not sweet at pH 7 but is sweet at lower pH. The crystal structure of neoculin at neutral condition was determined in 2006 [9] and it showed an oligomeric structure comprising four monomers. Neither homodimer, NAS or NBS alone, shows any sweetness at either neutral or acidic pH.

The three-dimensional structure of miraculin has not yet been reported. However, the reduced monomeric form of miraculin also shows no sweetness at any pH. Among the results from several mutational experiments on neoculin, an His11Ala mutant showed sweetness under both neutral and acidic conditions [10], while a Gln90Lys mutant showed sweetness at neutral conditions but reduced sweetness at low pH [11]. Thus, these two residues are thought to be important for pH-dependent properties but not for the receptor activation. The two residues do not directly interact since they are located far from each other in the monomer and show no hydrogen-bond partnering that could affect the pH dependence. Although these residues should contribute to the structural change of the protein to form a structure for binding and activating the sweet receptor, it remains unclear how proteins form an activated structure.

Neoculin is proposed to take on a “closed” form at neutral pH in the crystal structure but an “open” form at acidic pH based on the results of molecular dynamics (MD) calculations on neoculin in a virtual water system [9]. Here, we investigate the sweet taste-modifying properties of neoculin and its mutants in their dimeric or oligomeric structures by MD studies.

## Materials and Methods

**Fig 1:**
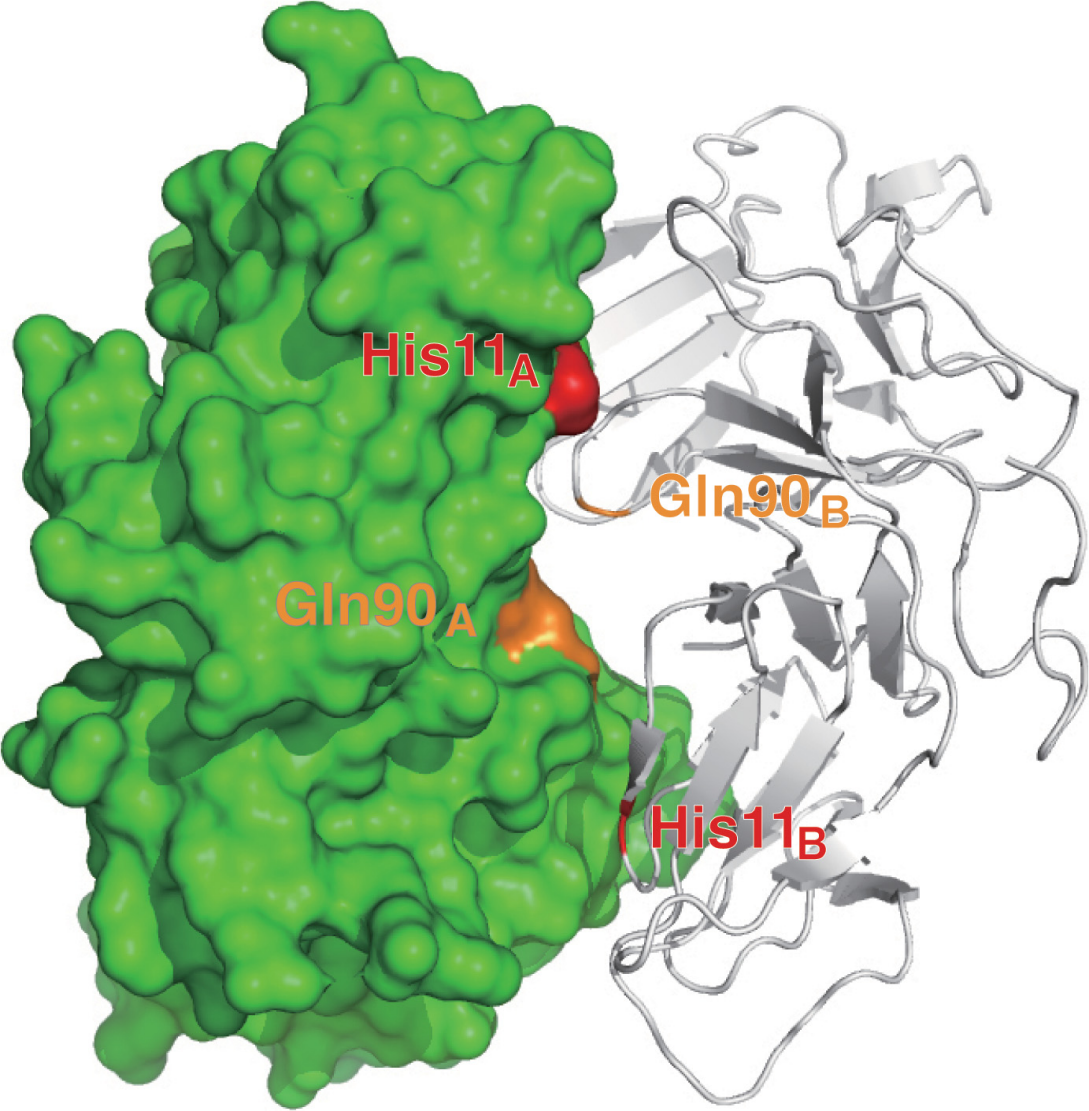
Crystal structure of the dimeric structure of neoculin. One neoculin molecule is depicted as a green surface with His11_A_ in red and Gln90_A_ in orange. The other neoculin monomer is represented with grey loop and ribbon structure.

The three-dimensional neoculin oligomeric structures (PDB code: 2D04) were used for modeling and molecular dynamics calculations. The neoculin crystal structure consists of four molecules, and among the four molecules examined, we selected a dimeric form in which a neoculin monomer forms a pseudosymmetric dimeric form with another neoculin monomer for further examination (Fig 1). In this dimeric structure, the His11 residue is located close to Asp91 in the other neoculin molecule at the interface of the two neoculin molecules while the Gln90 residue is also located at the interface but does not form a hydrogen bond with any residues. The initial structures of His11Tyr, His11Phe, His11Ala and Gln90Lys mutants were constructed by replacing each residue using the Homology module in molecular modeling software InsightII (Accelrys, Inc.). Wild type (WT) neoculin and each mutant were examined under neutral (pH 7) and acidic (pH 3) conditions. Under the acidic condition, Asp and Glu residues and the C-terminus of the protein were set to the protonated state (COOH), and His, Lys and Arg residues and the N-terminus of the protein were kept protonated. Under the neutral condition, Asp and Glu residues and the C-terminus of the protein were kept in the deprotonated state (COO^−^), and Lys and Arg residues and the N-terminus of the protein were kept protonated, while the His residues of the protein were kept deprotonated. The WT and mutants were solvated in a truncated octahedral TIP3P water box with a thickness of 8 Å around the protein with a force field of ff99SB under neutral and acidic conditions. The systems were neutralized by adding Cl^−^ions as a counter ion and the energy minimized (MM) and MD calculations were determined by using AMBER11/SANDER [12] with periodic boundary conditions with constant temperature (300 K) and pressure (1 atm) conditions and a non-bonded interaction cut-off distance of 14 Å. The particle mesh Ewald (PME) method was employed for electrostatic interaction calculations. The MM calculations of the system were performed in 1000 steps by the steepest descent method and 5000 steps by the conjugated gradient method with tethering of the heavy atoms of the protein. Then, MM calculations were performed in 1000 steps by the steepest descent method and 5000 steps by the conjugated gradient method for all of the atoms. Subsequently, MD calculations were performed only for water molecules in the system in 50 ps timeframe while increasing the temperature from 0 to 300 K. MM calculations and MD calculations were then performed for the whole system. After all atoms were heated from 0 to 300 K in 50 ps intervals for the MD calculations, the whole system was equilibrated for 10 ns for WT and the mutants at constant temperature (300 K) and pressure (1 atm).

Structural changes were evaluated based on the root mean square deviation (RMSD) and the distance between the centroids of each monomer in the trajectory of intermediate structures in the MD calculations to evaluate the distances between the two neoculin monomers.

## Results

MD calculations were performed at neutral (pH 7) and acidic (pH 3) conditions for the dimeric structure of neoculin (Fig 1), considering that neoculin elicits its sweetness in an aqueous solution of citric acid (pKa 2.87) [10]. The temperature and pressure were maintained to be constant. RMSD and the distance between centroids of two neoculin molecules calculated over the temperature range from 0 to 300 K with 500 ps MD calculations showed no significant dissociations at both conditions. Thus, at an early stage in the equilibration of MD calculations, the structures of the dimers maintained the dimeric interface and their initial monomeric structures.

**Fig 2:**
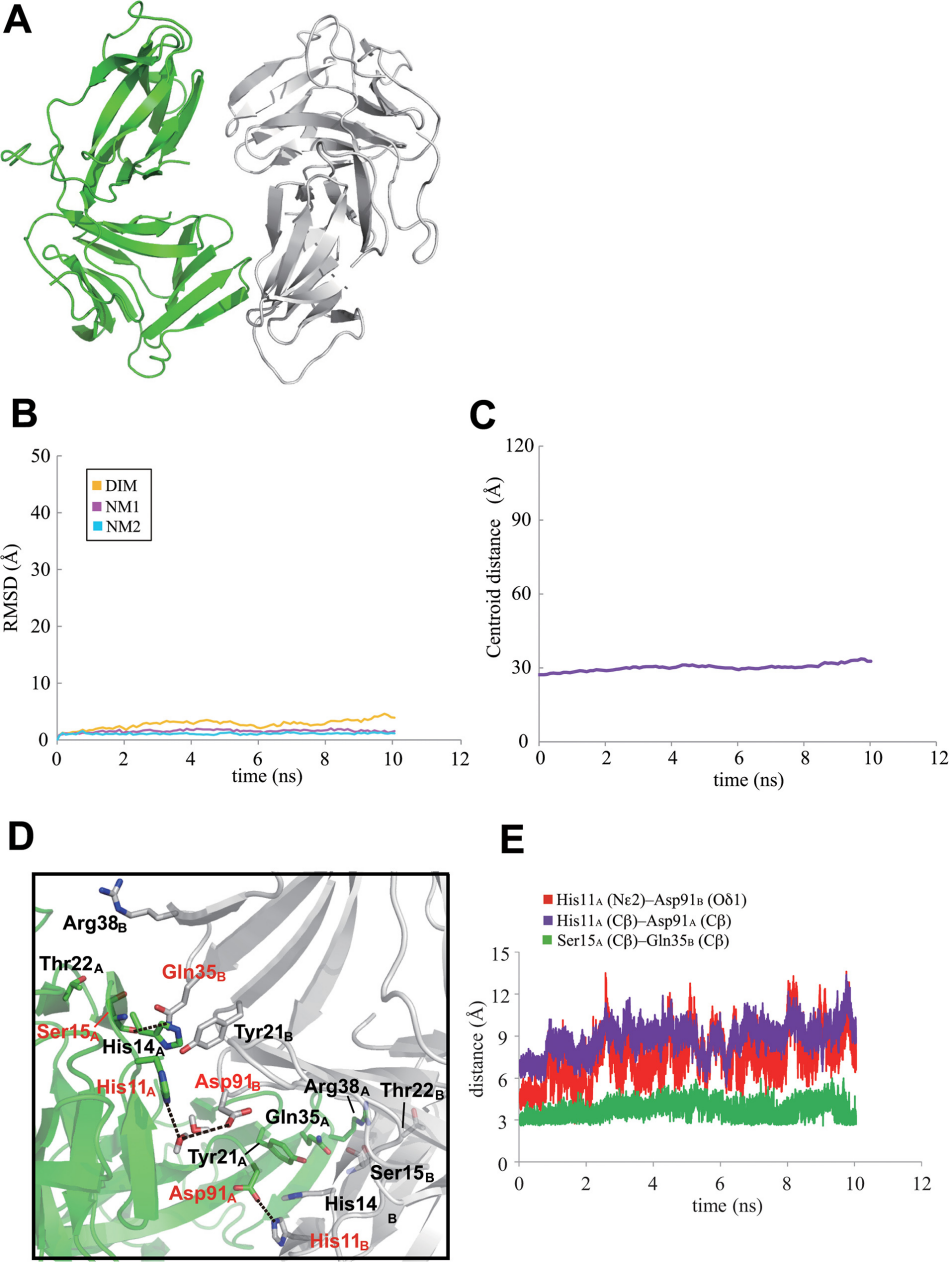
Dimeric structure of two neoculin molecules based on MD calculations at neutral pH after 10 ns. A. Two molecules are colored in green and grey. B. RMSD values for the main chain atoms of neoculin from the WT in the 10 ns MD trajectory. Orange, magenta, and blue lines represent the whole dimeric structure (DIM), one neoculin monomer (NM1), and the other neoculin monomer (NM2), respectively. C. Distance between the centroids of each monomer in the MD trajectory. D. Hydrogen bonds (dotted lines) at the interface. E. Time dependent distances between His11_A_ and Asp91_B_, His11_A_ (Cβ) and Asp91_B_ (Cβ) and Ser15_A_ and Gln35_B_ of WT neoculin in red, purple and green traces, respectively.

At the neutral condition and with 10 ns MD calculations, the initial structures of the neoculin monomers in the dimeric structure were maintained without major structural changes, keeping the dimeric structures at a neutral condition (Fig 2A) judging from the RMSD in the trajectory of the intermediate structures (Fig 2B) and the distance between the centroids of each monomer in the MD calculations (Fig 2C). As shown in Fig 2D, His11_B_ (subscripts _A_ and _B_ are assigned to each of the monomers in neoculin) maintained the hydrogen bond with Asp91_A_ in the dimeric structure, while a water molecule mediated the interaction between residues His11_A_ and Asp91_B_. Fig 2E shows a steady maintenance of the distance between His11_A_ and Asp91_B_ in the trajectory of the 10 ns MD calculation. Thus, the hydrogen bonds between His11 and Asp91 at the dimeric interface may contribute to the maintenance of the dimeric structure of neoculin at neutral pH.

Inspection of the interface of the dimer in the crystal structure revealed four additional electronic interactions. Of these, the hydrogen bond between the main chain carbonyl Ser15_A_ and the side chain amide Gln35_B_ was strong (2.64 Å), while the other three interactions were weak: the side chain carbonyl of Gln35_A_ and the main chain nitrogen of Ser15_B_ (3.29 Å), the hydroxyl oxygen of Thr22_A_ and the side chain nitrogen (Nη) of Arg38_B_ (3.46 Å), and the side chain nitrogen (Nη) of Arg38_A_ and the hydroxyl oxygen of Thr22_B_ (3.31 Å). The distances between Ser15_A_ and Gln35_B_ in the trajectory of the MD calculation indicates steady maintenance of the hydrogen bonds between them (Fig 2E). Since significant hydrophobic interactions (i.e., contact between hydrophobic residues) were not found, the interactions appear to be maintained by electronic interactions (hydrogen bonds), as depicted in Fig 2D.

**Fig 3:**
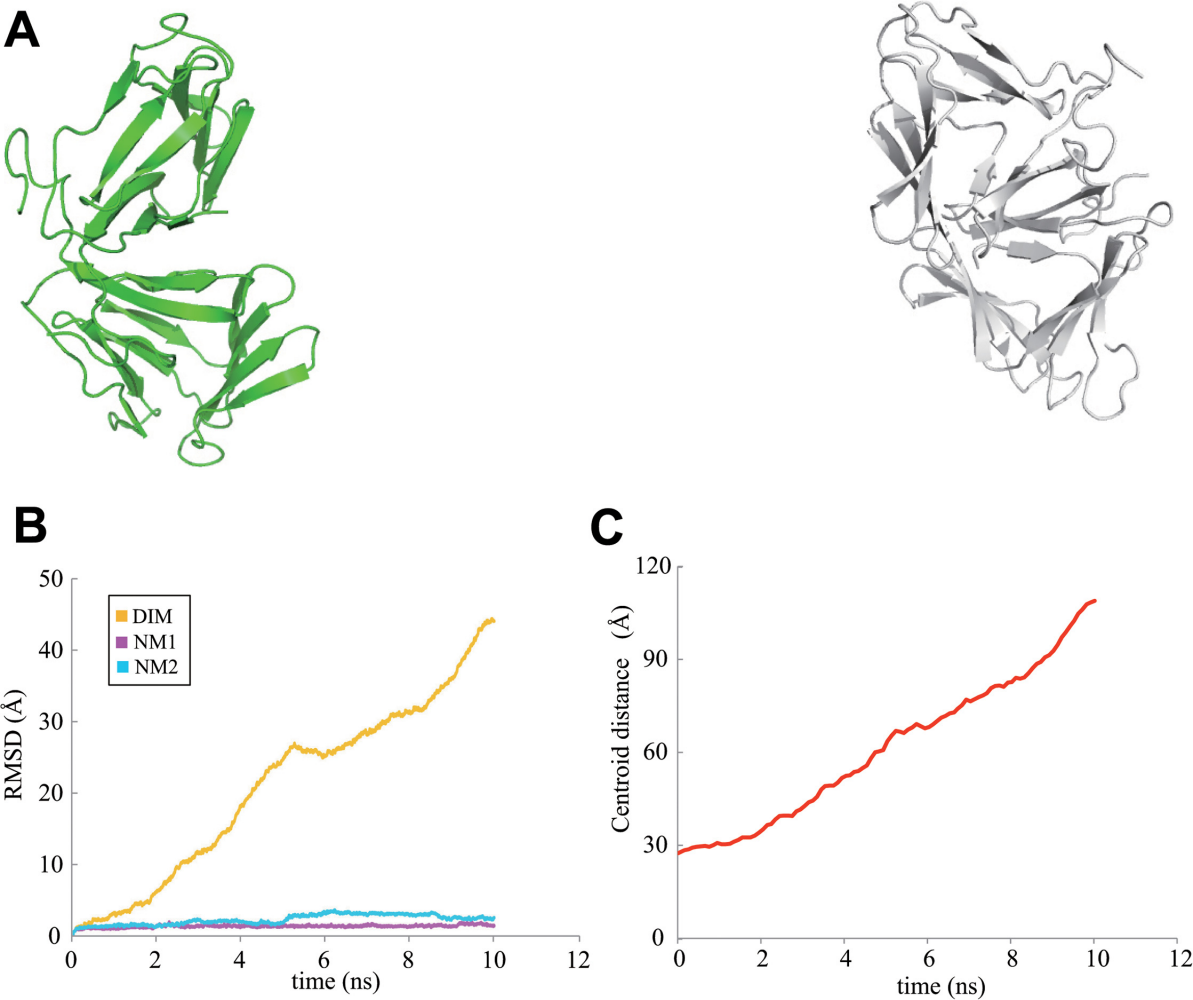
The dissociated neoculin monomers after MD calculations at pH 3. A. Dissociated structures after 10 ns. B. RMSD values for the main chain atoms of neoculin in the 10 ns MD trajectory from the WT. Line color is as defined for Fig 2B. C. Distance between the centroids of each monomer in the MD trajectory.

The MD calculations showed no great structural changes for each neoculin monomer under the acidic condition. However, the initial dimeric protein greatly dissociated to produce isolated monomers of the protein (Fig 3A) as demonstrated by the increasing RMSD of the dimer and the distance between the centroids of each monomer (Fig 3B and 3C). The protonated His11_A_ residue could not form a hydrogen bond with the protonated Asp91_B_ residue in the dimeric structure, and these two residues could not form a hydrogen bond with any other residues in the other monomer for the conditions used in the 10 ns MD calculation.

**Fig 4:**
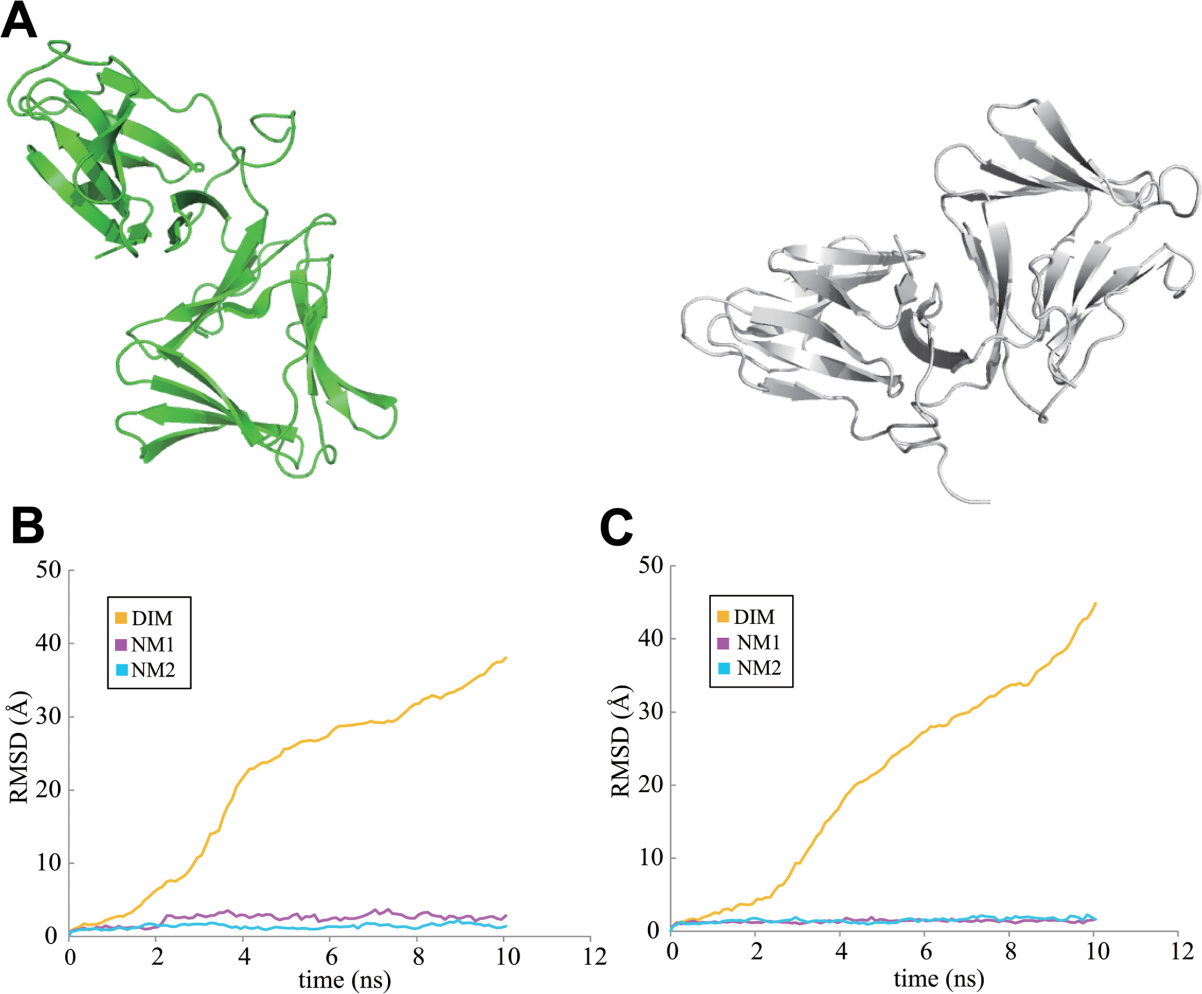
The dissociated monomers of neoculin His11Ala mutant. A. Dissociated structures after MD calculations under neutral conditions. B. RMSD values for the main chain atoms of the neoculin mutant in the 10 ns MD trajectory compared to the initial structure (MD under the neutral pH condition). C. RMSD values for the main chain atoms of the neoculin mutant in the MD trajectory from the initial structure (MD under the acidic condition). Line color is as defined for Fig 2B.

MD behaviors of His11 mutants have been studied under the same conditions as for WT neoculin. The MD calculations on a His11Ala mutant dimer model were performed under neutral and acidic conditions. The dimer dissociated to produce isolated monomers regardless of neutral or acidic conditions (Fig 4A). RMSD analysis indicated that each monomer maintained the initial structure derived from WT, while the dimer dissociated to give each isolated monomer (Fig 4B and 4C). The substitution of Ala for His11 disrupted the original His11-Asp91 interaction found in the WT neoculin dimer and, thus, promoted the dissociation of the monomers. The disappearance of the hydrogen bond between the dimeric structures is expected to weaken the interactions at the interface of the dimer.

**Fig 5:**
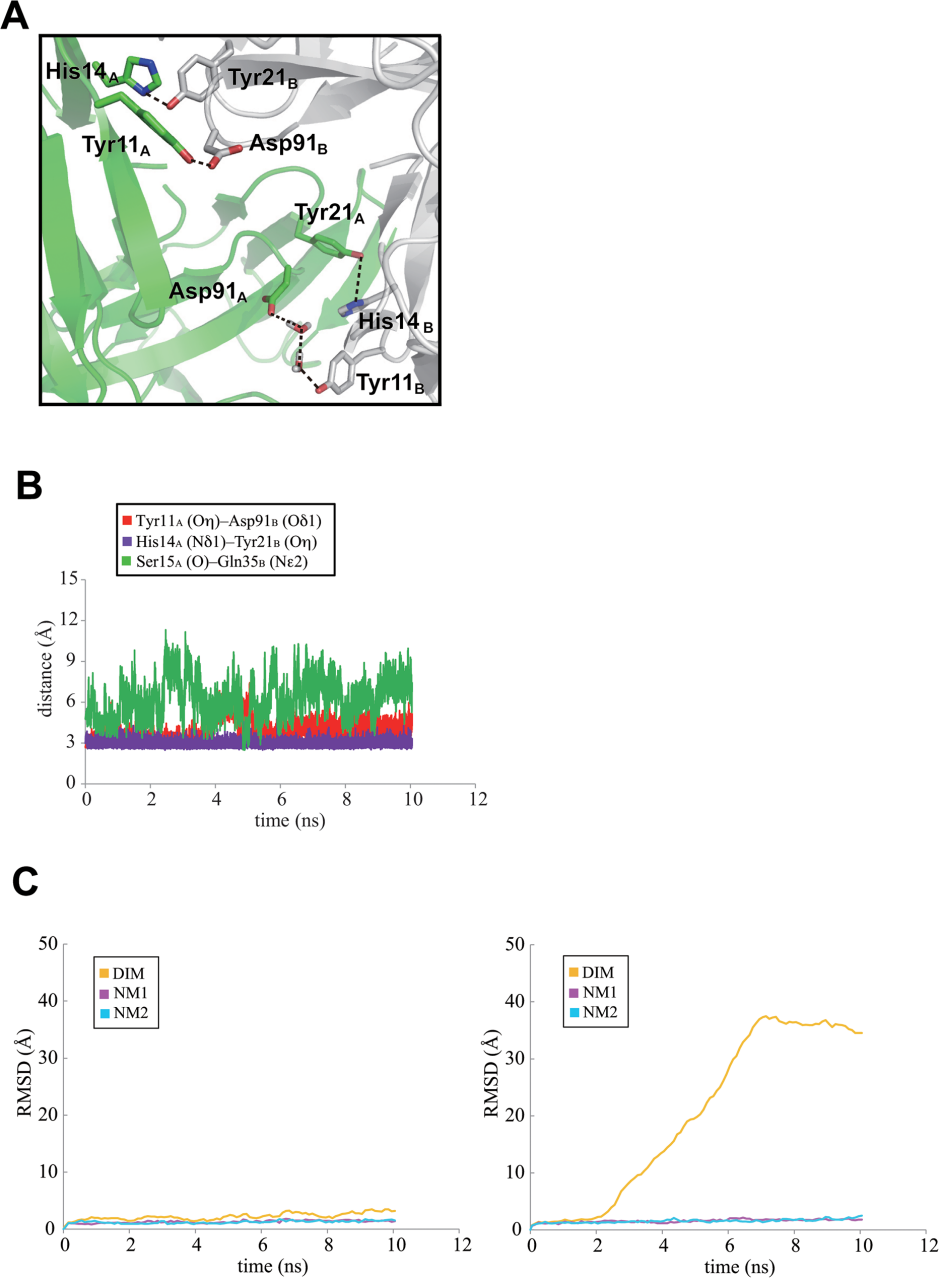
Dimeric structure of His11Tyr mutant under neutral condition. A. Hydrogen bond interactions shown as dotted lines at the interface. B. Time dependent distances between Tyr11_A_ and Asp91_B_, His14_A_ and Tyr21_B_ and Ser15_A_ and Gln35_B_ of the mutant under neutral conditions in red, purple and green traces, respectively. C. RMSD values for the main chain atoms of the His11Tyr mutant the 10 ns MD trajectory from the initial structure under the neutral condition (left) and the acidic condition (right). Line color is as defined for Fig 2B.

The His11Tyr mutant lost most of its sweetness under the neutral condition, while the His11Phe mutant maintained weak sweetness at a level similar to that in the WT [10]. The two mutants showed sweetness as strong as WT under the acidic condition. With the replacement of His11 residue with Tyr or Phe, determination of MD behaviors of these mutants under the same conditions as applied to WT demonstrated that the His11Tyr mutant did not change its dimeric structure under neutral conditions but was similar to that for WT and showed a shorter hydrogen bond (2.68 Å) between Tyr11_A_ and Asp91_B_ than the hydrogen bond of His11-Asp91 (2.85 Å) in the WT dimeric structure. In addition, a new hydrogen bond was found between His14_A_ and Tyr21_B_ at the interface (Fig 5A). As shown in Fig 5B, the mutant steadily formed the hydrogen bond throughout the MD calculation. This hydrogen bond was not found in the WT and the His11Ala mutant structures. These hydrogen bonds (Tyr11-Asp91 and His14-Tyr21) suggest a stronger interaction at the interface under conditions of neutral pH. On the other hand, MD calculations under the acidic condition led to dissociation of the dimer to the isolated monomers (Fig 5C), suggesting that neither protonated Asp91 nor His14 is a good hydrogen bond acceptor or donor for Tyr11 or Tyr21, respectively, which is necessary in order to maintain the dimeric structure.

**Fig 6:**
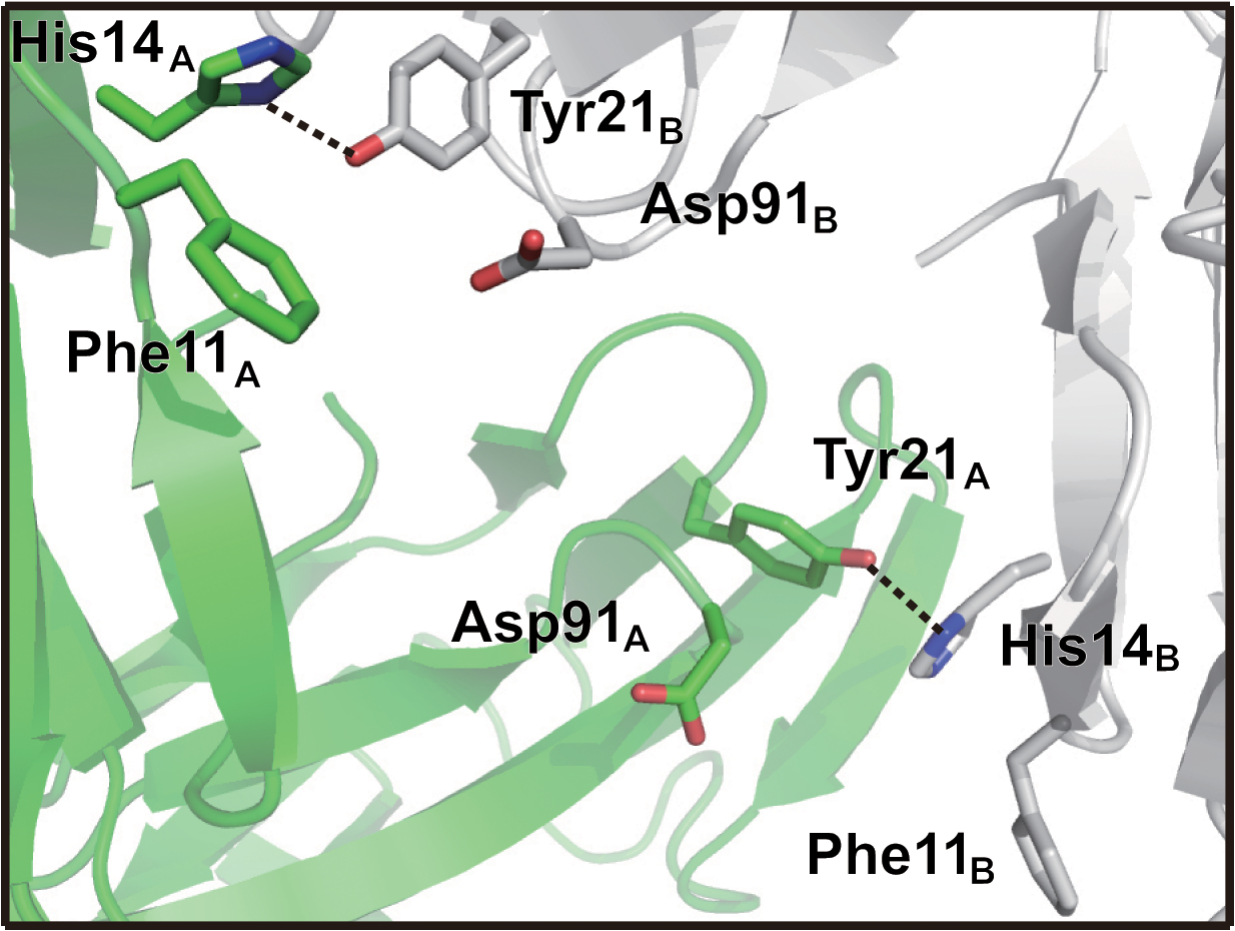
Dimeric interface of His11Phe mutant. Dotted lines show hydrogen bonds at the interface.

**Fig 7:**
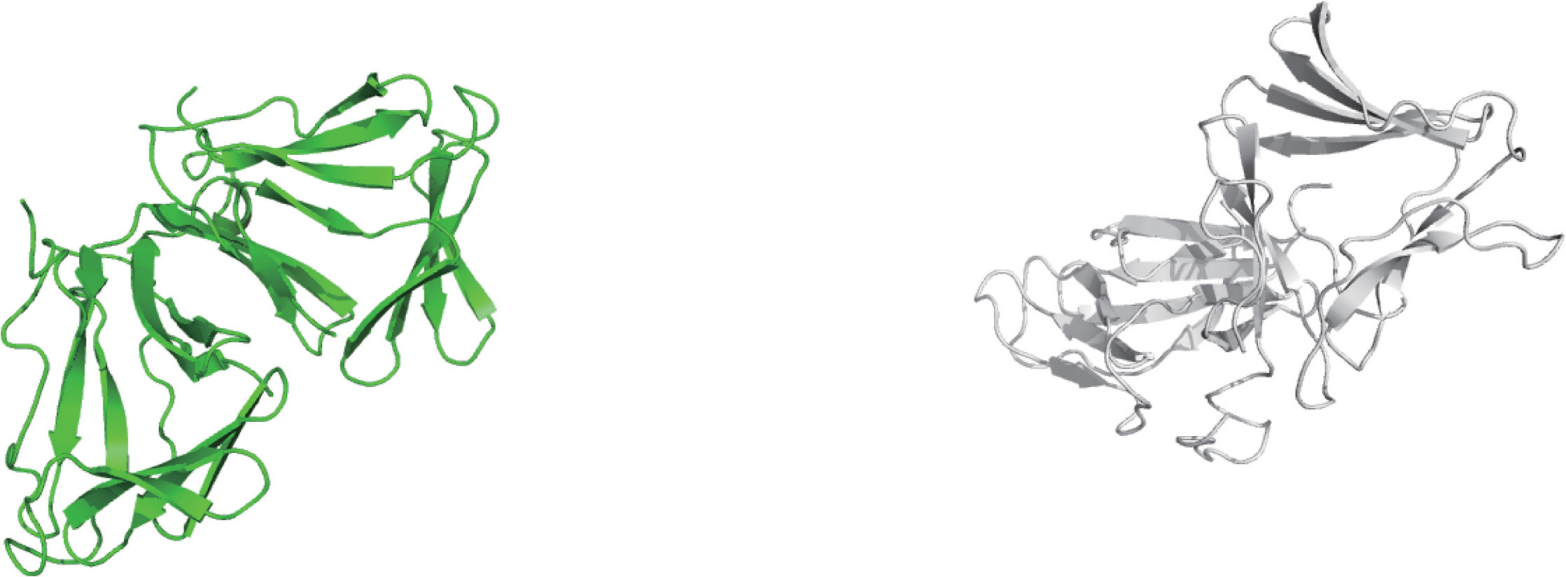
Dissociated monomers of the His11Phe mutant in MD calculations under the acidic condition.

The Phe11 residue in the His11Phe mutant could not form a hydrogen bond with Asp91 at the interface of the dimer. The MD calculations for the model of the His11Phe mutant showed no significant dissociation of the dimer (Fig 6). The Phe11 residue of the mutant was not located in a position that suggests appreciable hydrophobic or aromatic-aromatic interactions with residues in other neoculin molecules. However, another favorable interaction between His14_A_ and Tyr21_B_ was found at the dimeric interface, as found in the His11Tyr mutant. Thus, this newly formed hydrogen bond may become a substitute for the His11-Asp91 interaction, contributing to the stabilization of the dimeric structure of the His11Phe mutant under the neutral condition. The acidic condition that caused the His14 residue to switch to the protonated state weakens the hydrogen bond between His14 and Tyr21 to dissociate the dimeric structure (Fig 7) as observed in the WT and His11Tyr and His11Ala mutants.

**Fig 8:**
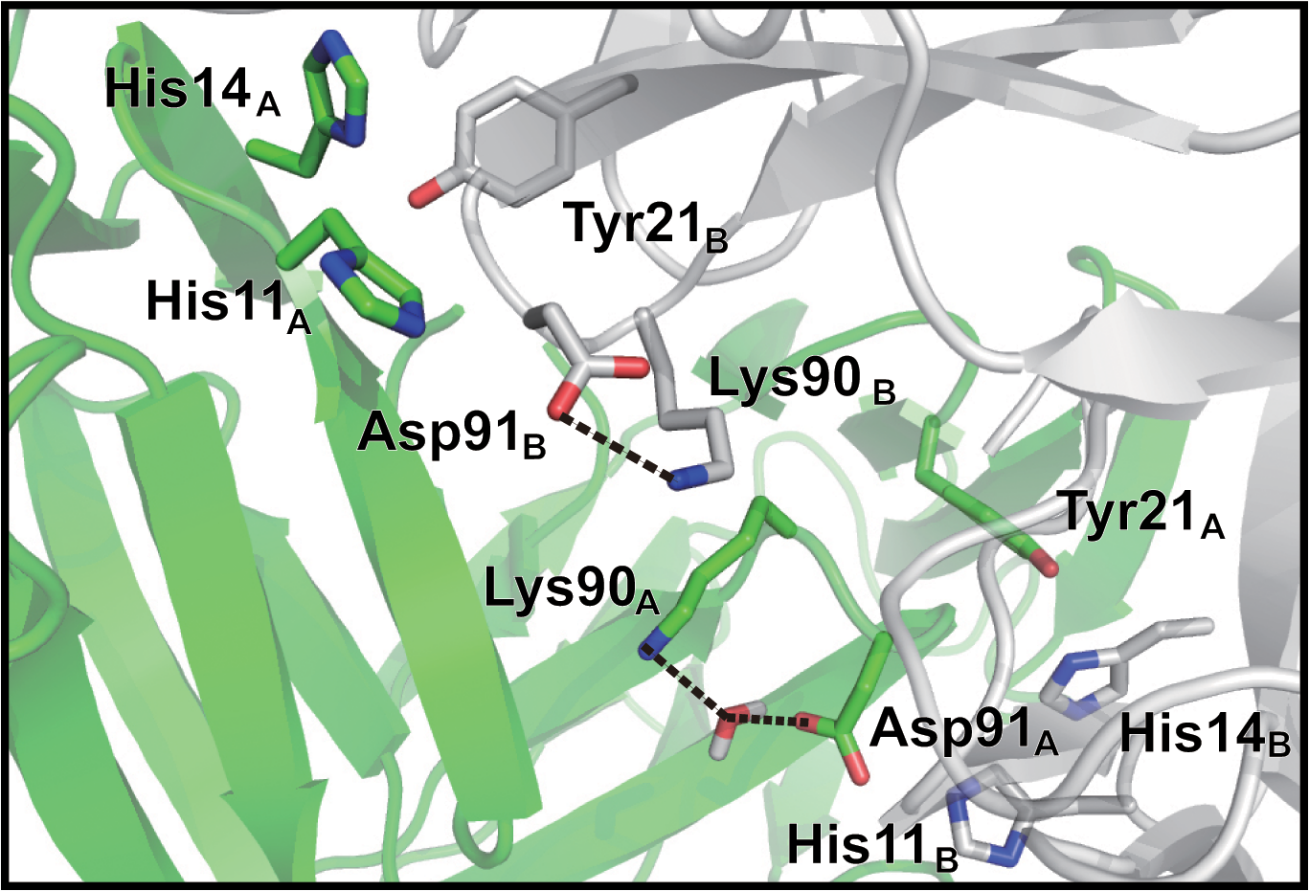
Hydrogen bond interactions of the Gln90Lys mutant under the neutral condition. Two initial structures of monomers at the interface.

**Fig 9:**
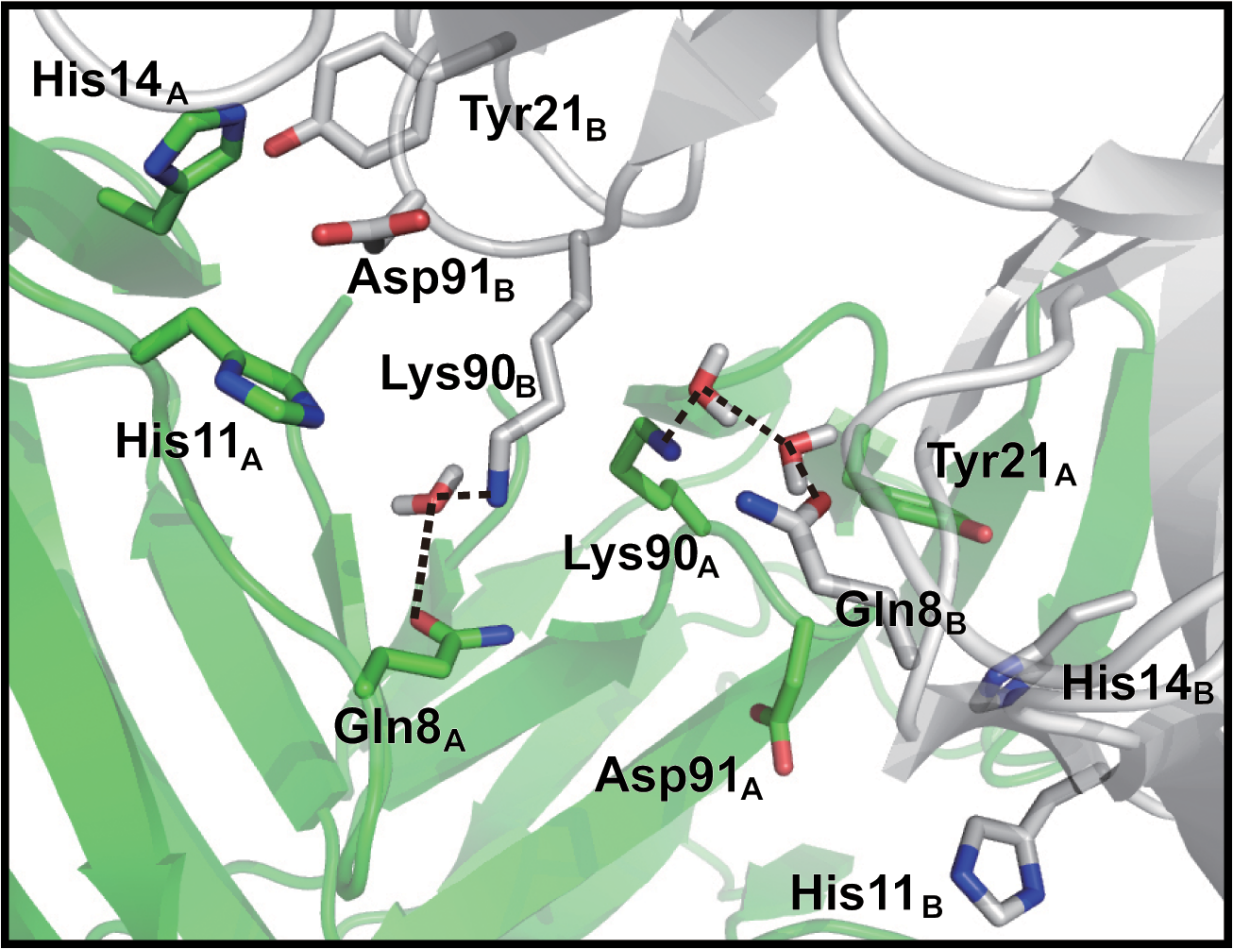
Hydrogen bond network at the dimeric interface of the Gln90Lys mutant under the acidic condition.

As reported by Kurimoto *et al*., the Gln90Lys mutant showed sweetness properties that were opposite to those of WT [11]. This mutant shows stronger sweetness than WT under the neutral condition and loses sweetness under the acidic condition. An initial model of the Gln90Lys mutant under the neutral condition (Fig 8) indicated the formation of a hydrogen bond between Lys90_B_ and neighboring Asp91_B_ in the same monomer and this interaction disrupted the hydrogen bond between Asp91_B_ and His11_A_ at the interface. Thus, the Asp91_B_ residue switched its hydrogen bond partner from His11_A_ to neighboring Lys90_B_ (Fig 8). As a result, the MD calculations under the neutral condition resulted in the dissociation of the dimer of the mutant. On the other hand, the protonated Asp91_B_ residue formed a hydrogen bond with neither Lys90_B_ nor His11_A_ under the acidic condition. Instead, the Lys90_B_ residue formed a hydrogen bond with Gln8_A_ of the other neoculin monomer (Fig 9). Thus, the Gln90Lys mutant maintained its dimeric structure during the MD calculations under the acidic condition.

**Fig 10:**
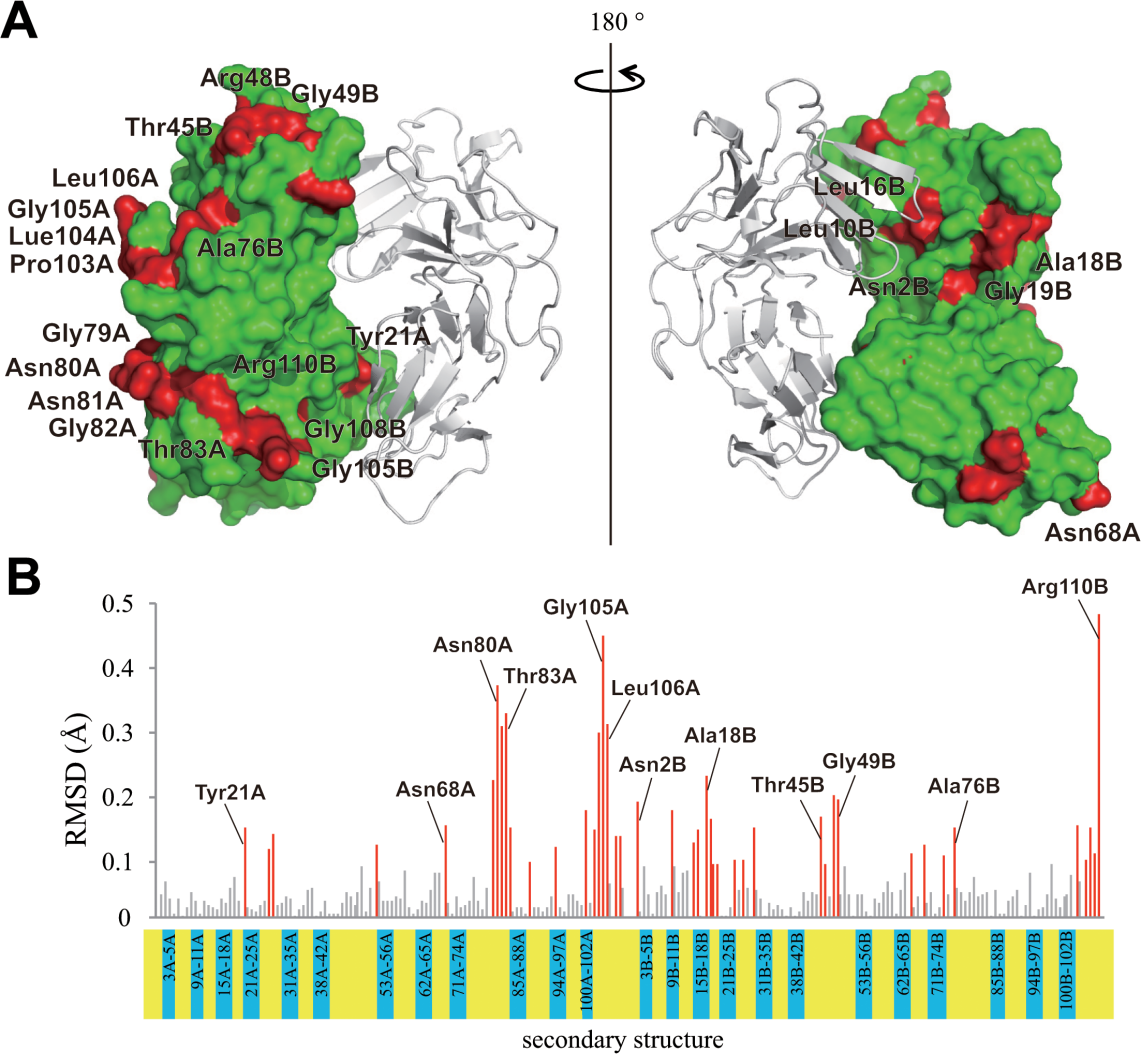
Location of residues showing mobile main chains on the neoculin monomer. A. The mobile residues are colored in red based on the difference in RMSD value for the main chain atoms of each residue in the MD trajectory. Residues in NAS and NBS are indicated by A and B with the residue number, respectively. B. The difference in RMSD value for the main chain atoms of each residue in the MD trajectory. Bars for mobile residues with RMSD values of ≥0.1 are colored in red and immobile residues with RMSD values of <0.1 are colored in grey. The regions of secondary structures are indicated in blue below the graph, and the yellow-colored regions are loop structures.

**Fig 11:**
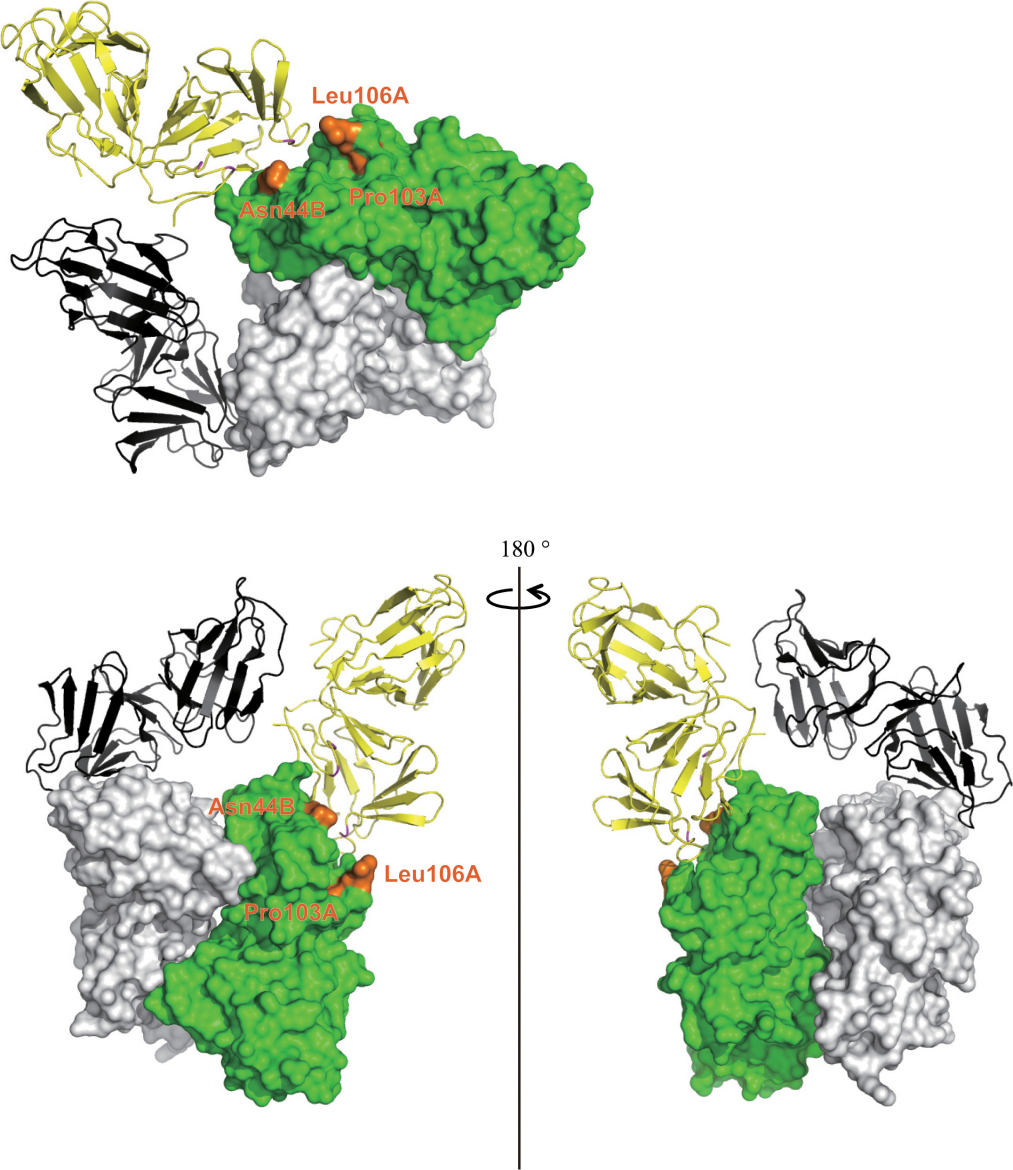
Tetrameric structure of neoculin monomers in the crystal structure. A. The surfaces of Pro103A, Leu106A and Asn44B are colored orange. Another monomer (yellow ribbon) has contact with neoculin near these three residues. The other two neoculin molecules are shown as a grey surface and a black ribbon. B. Tetrameric structure rotated 90° clockwise from the structure depicted in A (left). Rotation of the structure on the left 180° about a vertical axis to show the structures on the other side of the dimer (right).

**Fig 12:**
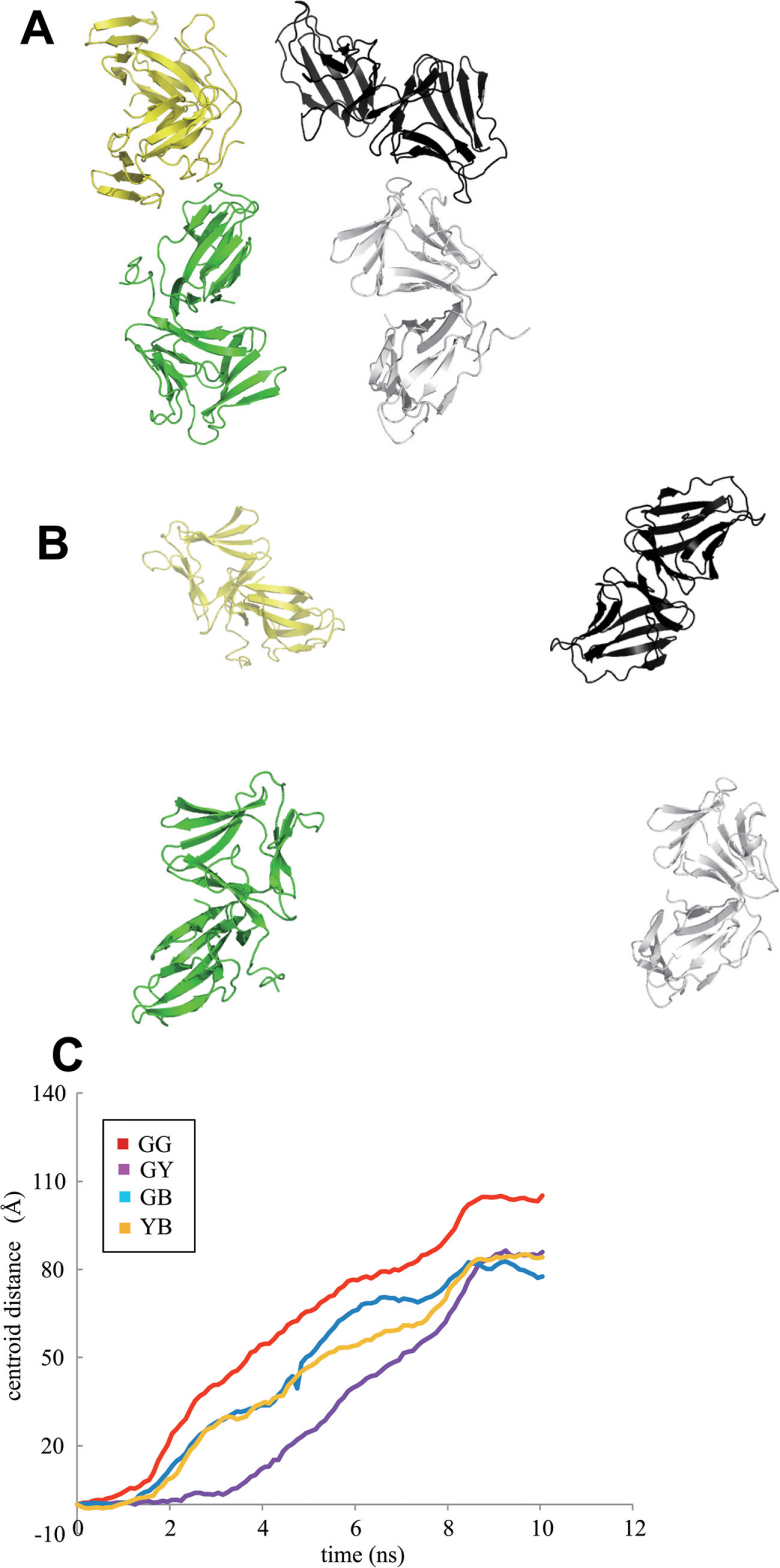
Dissociation of neoculin oligomer in MD calculations at acidic pH. A. An early stage (2 ns). B. A late stage (10 ns). Line color is as defined for Fig 11. C. Distance between the centroids of each monomer in the 10 ns MD trajectory. Red, blue, purple and orange lines to represent the centroid distances between pairs of monomers represented in green and grey (GG), green and black (GB), green and yellow (GY), and yellow and black (YB), respectively.

The dissociated monomer of WT neoculin showed locally more flexible main chains than for the dimer based on RMSD values between the trajectory of the corresponding main chains under the neutral and acidic conditions (Fig 10A). These locally flexible sites are located at the loop structures (Fig 10B) and are not involved in the dimeric interface as are other sites where other monomers interact with the oligomeric structures as shown in Fig 11. Thus, MD calculations were performed on the oligomeric structure (the tetramer) found in the crystal structure. On a snapshot of the intermediate stage in the MD calculations (at 2 ns; Fig 12A), the dissociation at the interface where His11 was involved at an earlier stage is shown, along with the maintenance of the dimeric structure of the other interface with the other monomer near residues Pro103, Leu106 in NAS and Asn44 in the NBS, which may be involved in interactions with the sweet receptor [11]. Fig 12B shows the final stage at 10 ns in which the oligomer is dissociated to the four isolated neoculin monomers. The trajectory of the RMSD in the MD calculations also supports this described dissociation pattern (Fig 12C). Dissociation at the early stage may be governed by the change in protonation states of the residues at the interface, and the following dissociation at the later stage may be promoted by local motion at the other interface of the neoculin monomer in which no ionic interactions are involved.

## Discussion

The mutational experiments reported by Nakajima *et al* [10] indicated that His11 plays an important role in the pH dependence of the sweetness-modifying activity of neoculin. Here, we have performed MD calculations on the crystal structure of the WT form and models of several neoculin mutants. As described in the results of the calculation on the dimeric structures of the WT and each of the mutants, each neoculin monomer of the dimers did not show significant structural changes. Thus, the taste-modifying property was not ascribed to structural changes of the neoculin monomers. The inspection of the crystal structure of neoculin in the protein data bank indicated that neoculin forms an oligomeric structure. Among the oligomeric structures (four molecules in a unit cell), the pseudosymmetric dimer (Fig 1) was found for His11 located at the interface. Thus, we have examined the interactions of His11 with residues in the other neoculin molecules under neutral and acidic conditions using MD calculations under aqueous conditions. The WT neoculin maintained a dimeric structure at pH 7, but it promptly dissociated to isolated monomers at pH 3, as shown in Fig 3A. This result suggests that the monomeric structure at pH 3 is essential for the sweetness activity of neoculin, but that the dimer form may not elicit receptor activation. The His11 residue may contribute to the formation of the dimeric structure under neutral conditions. Since Ala11 of the His11Ala mutant makes no contribution to hydrogen bond formation at the interface, the mutant could become a monomer as shown in Fig 4A and show the sweetness activity irrespective of pH [10]. It is interesting that the His11Phe mutant showed a similar taste-modifying property as neoculin, despite the lack of a hydrophilic group on the phenyl group. The MD calculations provided new information about the hydrogen bond interactions at the interface. Due to the bulky phenyl group, Phe11 caused conformational changes in the residues around the Phe11 residue that induced a hydrogen bond between His14_A_ and Tyr21_B_ (Fig 6). This interaction was not observed in the WT or the His11Ala mutant. Thus, the new hydrogen bond may contribute to maintaining the dimeric structure under the neutral pH condition. Under the acidic condition, the hydrogen bond between His14_A_ and Tyr21_B_ should become weak and, thus, the dimer should dissociate to form isolated monomers similar to WT neoculin. On the other hand, the His11Tyr mutant showed a stronger hydrogen bond than for the WT between Tyr11_A_ and Asp91_B_ (Fig 5A). Moreover, His14_A_ of this mutant formed a hydrogen bond with Tyr21_B_ as shown in the His11Phe mutant. Formation of these two hydrogen bonds should lead to a tighter dimeric structure that is expected to make the mutant dimer less sweet under the neutral condition [10]. These two hydrogen bonds are expected to be lost under the acidic condition, thus enhancing monomer formation and making the mutant as sweet as neoculin. Thus, the residues bearing a phenyl ring make a unique contribution to the stability of the dimeric structure of neoculin under the neutral condition. It is interesting that one or two hydrogen bonds control the dimeric form. It follows that neoculin is slightly sweet at pH 7, as the inactive form of neoculin equilibrates with an active sweet form under the neutral condition. There is no significant hydrophobic interaction at the interface and, thus, the hydrogen bonds may govern the stability of the dimeric structure.

There is another mutant that does not have a mutation at the His11 residue but at the Gln90 residue. Gln90_B_ is located next to Asp91_B_, which along with Lys90_B_ of the mutant forms a hydrogen bond with Asp91_B_ in the initially energy-minimized structure and thus Lys90_B_ interrupts the hydrogen bond between Asp91_B_ and His11_A_ (Fig 8). The MD calculations indicate that the mutant dissociates to the isolated monomer under the neutral condition. Thus, the mutation affects the role of His11, which acts to stabilize the dimeric structure formation at the interface, suggesting that the Gln90Lys mutant would show sweetness under the neutral condition. Nakajima *et al*. reported that neoculin has a strong sweet taste at pH 6.3 in the presence of 100 mM ammonium chloride or at pH 8.0 in the presence of 100 mM ammonium acetate [13]. The effect of the ammonium ion would be similar to that of the role of the protonated Lys90 residue, which interrupts the His11-Asp91 interaction to make the mutant sweet at neutral pH. At acidic pH, the protonation of Asp91_B_ should weaken the hydrogen bond with Lys90_B_, and Lys90_B_ could then find another residue, Gln8_A_, on the other neoculin molecule with which to form a hydrogen bond at its interface (Fig 9). Thus, the hydrogen bond may maintain the dimeric structure under the acidic condition, resulting in reduced sweetness.

As mentioned previously, WT neoculin shows weak sweetness at neutral pH [10]. The present MD calculations suggest that the dimer and the isolated monomer are correlated to the taste-modifying properties of neoculin and its mutants under neutral and acidic conditions. Since the isolated monomer would be the minor form at the neutral pH, a small amount of isolated neoculin monomer may contribute to the weak sweetness. An increase in hydrogen bonding at the interface would reinforce dimer formation of the protein, leading to the inactivation observed in the His11Tyr mutant.

The dissociated monomer showed more flexible sites than the monomers fixed in the dimer locally. The flexible sites are not at the dimeric interface but at other sites where the other monomer interacts in the oligomeric structure (Fig 11). Kurimoto *et al*. have reported the sweetness activity for several mutants of neoculin in which non-conserved residues in NAS and NBS are mutated Pro103 and Leu106 in NAS and Asn44 in NBS, demonstrating the importance of these residues for receptor activation [11]. These residues are aligned in the same area and are localized to sites where the other monomer forms oligomers. Thus, the essential site for receptor binding/activation may be blocked by the other monomer in the oligomeric structure at neutral pH. The MD calculations on the oligomeric structure (four neoculin molecules) under acidic pH conditions showed that the interface involving His11 dissociates first (Fig 12A) followed by other molecules on the other interfaces to form the four isolated monomers (Fig 12B and 12C).

It has been shown that neoculin binds at the amino terminal domain (ATD) of T1R3 in the heterodimeric human T1R2/T1R3 [14]. The monomeric neoculin, which is comprised of the NAS and NBS heterodimeric domains, would recognize the ATD of human T1R3 with the region involving Pro103 and Leu106 in NAS and Asn44 in NBS and would cause structural changes to the activated structure of the receptor, as shown in the agonist-bound structure of the metabotropic glutamate receptor [15]. The present study findings may allow use of a monomeric crystal structure of neoculin for docking studies focused on the N-terminal extracellular domains (ATD and Cys-rich domain). It has been reported that neoculin shows antagonistic function at pH 7 [16]. However, the receptor-binding region of neoculin is not known, and it is not clear whether neoculin binds at T1R2, T1R3 or both. As discussed above, the putative region for the receptor activation is covered in the oligomeric structure under neutral conditions and, thus, regions of neoculin binding to the receptor could be different from those for receptor activation.

As mentioned earlier, the interface involving His11 is maintained by electronic interactions that are highly susceptible to changes in pH. The other interface involving Pro103 and Leu106 in NAS and Asn44 in NBS includes hydrophobic interactions, which would not be readily affected by changes in pH. This difference may be correlated to different dissociations of the monomers.

## Conclusions

We performed MD calculations on the dimeric and oligomeric structures of neoculin and its mutants under neutral and acidic conditions in a water system. The structure of the neoculin monomer did not show significant structural changes under neutral and acidic conditions, while the dimeric structure showed the dissociation of monomers at acidic pH. The neoculin mutants also showed dissociation of the dimeric structure; thus, the formation of isolated monomers is consistent with the observed sweetness. His11 of WT neoculin forms a hydrogen bond with Asp91 in another neoculin molecule. The protonation of His11 facilitates the dissociation of the oligomer at the interface. Thus, His11 plays an essential role in the pH dependence of the sweetness of neoculin. An important region for the receptor activation, Pro103 and Leu106 in NAS and Asn44 in NBS, is located far from the His11 region and under neutral conditions is blocked by the other neoculin monomer in the oligomeric structure of neoculin. The MD calculations on the tetrameric neoculin structure under acidic conditions showed that the interface involving His11 dissociates first and then the region for the receptor activation becomes more flexible, leading to a complete dissociation to generate the isolated monomers.

## Supporting Information

S1 Fig Time dependence of instantaneous temperature and total energy. Temperature dependence in the MD calculations on the WT neoculin dimer under neutral and acidic conditions is shown in red trace and total energy in blue one.
(EPS)

S2 Fig Time-evolution of relevant hydrogen bonds under acidic conditions. A. Time dependent distances between His11_A_ and Asp91_B_, His11_A_ (Cβ) and Asp91_B_ (Cβ) and Ser15_A_ and Gln35_B_ of WT neoculin in red, purple and green traces, respectively. B. The distances between Tyr11_A_ and Asp91_B_, His14_A_ and Tyr21_B_ and Ser15_A_ and Gln35_B_ of the His11Tyr mutant under acidic conditions in red, purple and green traces, respectively.
(EPS)
